# Cytotoxic Effects of Phytomediated Silver and Gold Nanoparticles Synthesised from Rooibos *(Aspalathus linearis*), and Aspalathin

**DOI:** 10.3390/plants10112460

**Published:** 2021-11-15

**Authors:** Akeem O. Akinfenwa, Naeem S. Abdul, Fathima T. Docrat, Jeanine L. Marnewick, Robbie C. Luckay, Ahmed A. Hussein

**Affiliations:** 1 Department of Chemistry, Cape Peninsula University of Technology, Bellville 7535, South Africa; oa.akeemlaja@gmail.com; 2 Applied Microbial and Health Biotechnology Institute, Cape Peninsula University of Technology, Bellville 7535, South Africa; sheikabduln@cput.ac.za (N.S.A.); docratt@cput.ac.za (F.T.D.); marnewickj@cput.ac.za (J.L.M.); 3 Chemistry & Polymer Science Department, Stellenbosch University, Matieland, Stellenbosch 7602, South Africa; rcluckay@sun.ac.za

**Keywords:** aspalathin, green rooibos, synthesis, gold, silver, nanoparticles, cancer, cellular uptake

## Abstract

The green chemistry approach has continuously been applied for the synthesis of functional nanomaterials to reduce waste, environmental hazards, and the use of toxic chemicals among other reasons. Bioactive natural compounds have been found great potential in this regard and are used to improve the stability, activity, and biodistribution of metal nanoparticles (MNPs). Aspalathin (ASP) from *Aspalathus linearis* (rooibos) has a well-defined pharmacological profile and functional groups capable of both reducing and capping agents in the synthesis of metallic nanoparticles (NP). This study provides the first report of the phytomediated synthesis of gold and silver nanoparticles (AuNPs/AgNPs) via ASP and the green rooibos (GR) extract. The study demonstrated a green chemistry approach to the biosynthesis of nanoparticles of GR-AuNPs, ASP-AuNPs, GR-AgNPs, and ASP-AgNPs. The results showed that GR and ASP could act both as reducing and stabilising agents in the formation of crystalline, with different shapes and dispersity of NPs in the ranges of 1.6–6.7 nm for AgNPs and 7.5–12.5 nm for the AuNPs. However, the ASP NPs were less stable in selected biogenic media compared to GR NPs and were later stabilised with polyethene glycol. The cytotoxicity studies showed that GR-AgNPs were the most cytotoxic against SH-SY5Y and HepG2 with IC_50_ 108.8 and 183.4 μg/mL, respectively. The cellular uptake analysis showed a high uptake of AuNPs and indicated that AgNPs of rooibos at a lower dose (1.3–1.5 μg/mL) is favourable for its anticancer potential. This study is a contribution to plant-mediated metallic nanoparticles using a pure single compound that can be further developed for targeted drug delivery for cancer cells treatments in the coming years.

## 1. Introduction

Cancer, a multistep disease involving rapid and uncontrol cell multiplication, is among the world-leading health challenges that have attracted global concern. The rapid division of cancer cells is often prompted by genetic impairment, which leads to abnormal cell cytosis in different parts of the body, such as in the brain, lungs, and liver, to name a few. The major cancer treatment is chemotherapy using a chemically synthesised drug from bulk platinum (II) complexes such as cisplatin, oxaliplatin, and carboplatin. The potential anticancer activities of the platinum complexes were attributed with a high affinity of the platinum to sulphur binding and the guanine bases of DNA [1]. Platinum-based drugs have recorded tremendous success by slowly inhibiting the growth of and blocking tumour cells. However, huge concerns have been raised about the serious side effects, including kidney and liver toxicity and hearing impairment associated with these drugs. Besides platinum, other biocompatible metal-based drugs such as gold and silver have been reported as promising anticancer agents [2,3]. As a result, strategies to reduce the side effects while improving the potencies of metal-based anticancer drugs have been ongoing. Among such strategies is the development of nanosystems for gold and silver as nanoparticles capped with antioxidant-rich plant sources for anticancer studies [4]. 

The plant-based synthesis method of metallic nanoparticles is considered less expensive and eco-friendly. The method involves the bio-reduction and stabilisation of metal NPs with phytochemical constituents of plants that act as reducing and capping agents. They are reportedly used as alternatives to synthetic drugs in the targeted delivery of metallic anticancer drugs due to their improved biodistribution and clearance in the body [5,6,7]. To expand the use of metals in nanomedicine, gold and silver functionalised with plant phytochemicals are commonly used for active drug delivery and diagnosis. The plant phytochemicals possess functional groups such as hydroxyl, amide, and sulphides that act as capping agents to enhance the activities of metal-based drugs through size reductions and increased surface areas [8]. Oyagi et al. (2014) previously reported the synthesis of nontoxic silver nanoparticles encapsulated by rooibos herbal tea components, which can be used for the targeted delivery of silver to cancer cells. In complementary research on rooibos, Diallo et al. (2016) demonstrated the biosynthesis of europium oxide nanocrystals from a rooibos extract, showing a luminescent property for potential application in the photothermal treatment of cancer cells [9,10].

*A. linearis* extracts have been reported in the synthesis of different metal NPs [9,10]; however, reports on the use of aspalathin (ASP) as a single stabilising and reducing agent in the plant-mediated synthesis of MNPs is still lacking. The phytochemical studies of rooibos revealed different flavonoids such as rutin, orientin, quercetin, luteolin, ASP, and many others to be present [11,12]. Aspalathin is a unique bio-antioxidant that has a *C*-glucoside dihydrochalcone skeleton and is found in high concentrations in green rooibos [13]. This compound is reported to play important roles in numerous health-promoting potentials of rooibos, including the protection of DNA strands and suppression of induced cancer growth [14,15,16]. The chemistry of ASP presents electron-rich and multi-binding sites favourable as a reducing agent in the synthesis of metallic nanoparticles. This property of ASP and the high phenolic content of rooibos extracts have been explored in different studies for biomedical applications. Still, limited information exists in the literature on the application of ASP for the synthesis of gold (Au) and silver (Ag) nanoparticles and the toxicity of the synthesised NPs on cancer cell lines. Although, the use of natural pure compounds such as hypoxoside, the proanthocyanidin dimer, and chalcones from different plant species in the preparation of Au- and AgNPs have been reported [17,18,19]. The current study presents a complimentary report on the synthesis of metal NPs using a single natural compound with a well-defined pharmacological profile, such as ASP. Hence, ASP, in addition to the total extract of *A. linearis* (GR), was used for the synthesis. Considering the importance of surface functionalisation with a well-defined pharmacologically natural compound is crucial to the bioactivity, biodistribution, and biocompatibility of different MNPs in the medical field; thus, this study aims to biosynthesise Au- and AgNPs capped with rooibos extract and pure ASP compound using a green chemistry approach to evaluate their cytotoxic activity against liver cancer and neuroblastoma human cell lines and determine the cellular uptake of the nanoparticles.

## 2. Results

**Figure 1:**
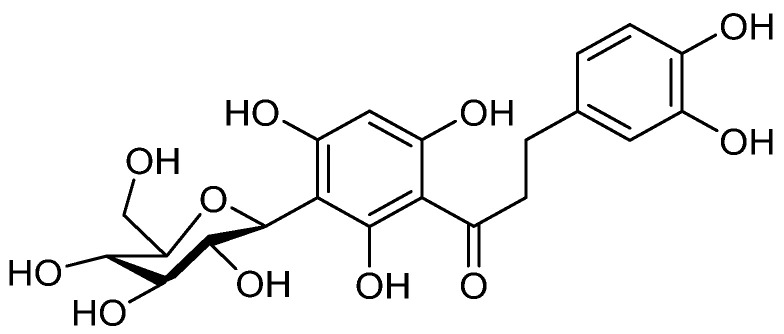
Chemical structure of aspalathin.

In continuation of the previous studies to probe the involvement of isolated compounds from plant extracts in the phytomediated synthesis of metallic nanoparticles [17,18,19], green rooibos (GR) and ASP have been selected as reducing and stabilising agents in this study because of their wide spectrum of biological activities and antioxidant potential. The isolation procedures of pure ASP (Figure 1) and its spectroscopic data has been reported recently [20].

### 2.1. Chemistry of Aspalathin

The chemistries of GR and ASP are interwoven, for GR was the only source of ASP until the time of this report. The chemical structure of ASP (Figure 1) makes it prone to oxidation due to its phenolic nature. This characteristic makes it suitable for use as a reducing agent that brings out the reduction of metals in solution to a stable zero valence state. Both GR and ASP demonstrated reducing and stabilising properties in the synthesis of both Au and Ag metal nanoparticles. 

### 2.2. UV–Vis Spectrophotometry Analysis

**Figure 2:**
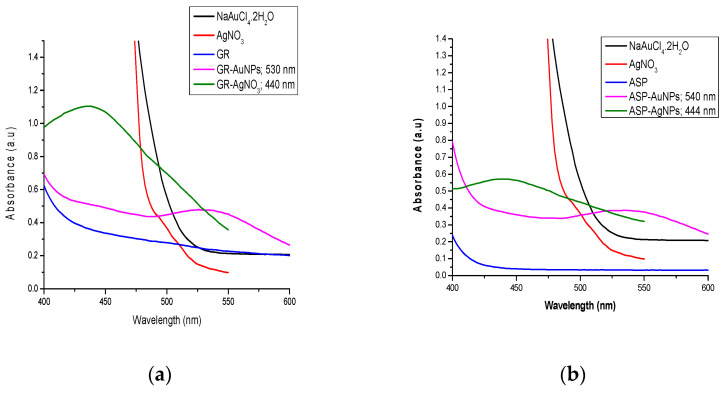
(**a**) UV–Vis spectra for the synthesised green Rooibos (GR) gold and silver nanoparticles. (**b**) UV–Vis spectra for the synthesised aspalathin (ASP) gold and silver nanoparticles.

The visual observation of colour change was quantified by the absorbance measurements for GR-AuNP, GR-AgNPs, ASP-AuNP, and ASP-Ag and presented in Figure 2a,b, respectively. Surface plasmon resonance (SPR) occurs due to the absorption of incident UV–Vis light by the solutions of transition metals. This phenomenon causes the surface electrons to collectively oscillate at a specific frequency. Au- and AgNPs are known to show characteristic SPR in the UV–Vis ranges of 500–600 (ruby red colour) nm and 400–500 nm (yellowish-brown), respectively. As noted in the Experimental section, the colour changes of the aqueous solutions of NaAuCl_4_·2H_2_O and AgNO_3_ at their corresponding absorptions maxima served as the initial indication of the formation of the NPs.

### 2.3. High-Resolution Transmission Electron Microscope Analysis 

**Figure 3:**
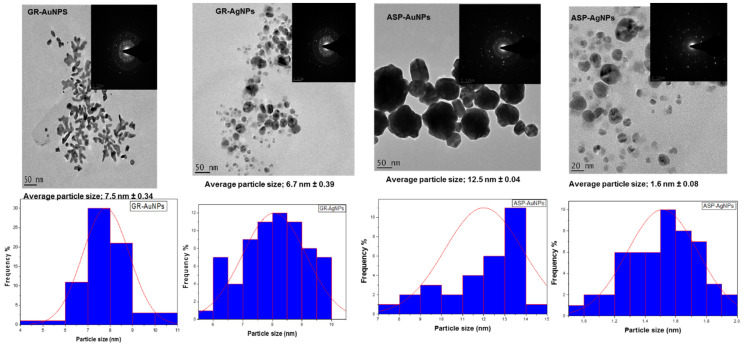
HRTEM images (left–right) for the GR-AuNPs, GR-AgNPs, ASP-AuNPs, and ASP-AgNPs. The corresponding insets are the SAED of the respective TEM images.

To confirm the morphology and size distribution of our NPs, TEM micrographs and selected area electron diffractions were recorded. The TEM images of all synthesised NPs revealed well-structured and non-agglomerated grains (Figure 3). The GR-AgNP and ASP-AgNP images displayed uniform quasi-spherical shapes in the size ranges of 6.7 ± 0.39 nm and 1.6 ± 0.08 nm, respectively. Several literature reports showed that the sizes and shapes of metal nanoparticles are influenced by different factors [21,22]. The synthesis procedure resulted in spherical sizes of the AgNPs that were in accord with the report by Oyagi et al. (2014). However, this study reports relatively bigger sizes of the AgNPs than was reported, which could be due to the differences in the rooibos types used in the studies [9]. The report of Analike et al. (2021) demonstrated the synthesis of mostly spherical AuNPs stabilised with gum arabic at 45 °C in the size range of 4.89–37.89 nm [23]. The AuNPs via GR and ASP showed rarely occurring hydra-like-shaped MNPs. The GR and ASP exhibited average size ranges of 7.5 ± 0.34 nm and 12.5 ± 0.04 nm, respectively. It is interesting to note that the GR NPs showed smaller particle sizes by two to three-fold than their corresponding ASP in each of the AuNPs and AgNPs. This observation is complementary to the several reports of the synergistic effects of the other compounds in GR during synthesis despite ASP being the major compound [17,18,19,24]. Remarkably, smaller sizes of GR and ASP-AgNPs were formed relative to the AuNP counterparts, even though both Au and Ag belong to the same family of *d*-block in the periodic table. These size distributions agree with the smaller ionic radii of silver when compared to gold.

### 2.4. Zeta Potential and Particle Size Distributions of Gold and Silver NPs

The DLS analysis of all NPs provided important information on the stability, size distribution, and the hydrodynamic diameters of the nanoparticles, as shown in Table 1. The zeta potential values (±mV) gave the degree of interparticle repulsion in a dispersion. It is an important factor to be considered for the stability of metallic NP to be used as a drug. Higher negative values greater than −25 mV are usually considered more stable. The GR-AuNPs (−26.2 mV) and ASP-AgNPs (−26.7 mV) were the most stable (Figure S1). The lower zeta potential values of −15.0 mV (GR-AgNPs) and −19.2 mV (ASP-AgNPs) were reported for biosynthesised AuNPs and AgNPs, which fell in the typical range of stable and non-agglomerated nanomaterials [7,25]. Furthermore, the polydispersity index (PDI) information obtained from the DLS represented the degree of homogeneity of different particle sizes to the total number of particles. The numerical values (no unit) for the PDI ranged from 0.0 to 1.0. Generally, particles with a PDI value of 0.0 were considered perfectly monodispersed. Values lower than 0.5 were accepted and indicated a near-homogeneous and monodisperse particle size distribution, while values above 0.5 tended towards the polydisperse limits [26]. As shown in Table 1 (Figure S1) the range of values of the PDI for all the NPs was indicative of near-uniform particle sizes showing the polydispersity index of the ASP-AgNPs (0.1), ASP-AuNPs (0.111), GR-AuNPs (0.128), and GR-AgNPs (0.274). The hydrodynamic sizes from the DLS measurements were the particles sizes in the colloidal system and were often higher than the mean particle size from TEM due to the attraction of surface charges of ions of opposite charges in the dispersed medium [18]. Evidently, (Figure S2) the hydrodynamic sizes of all the NPs were higher than the statistical calculations from the TEM images that supported the trend of the size variations in NPs of gold and silver from GR and ASP.

### 2.5. X-ray Diffraction (XRD) Analysis

We further confirm the crystalline structure of the Au- and AgNPs from GR and ASP using X-ray crystallography at room temperature. The AuNPs for both GR and ASP revealed four distinct Bragg peaks centred at 38.185, 44.393, 64.578, and 77.549 (2θ) corresponding to the crystallographic reflections (111), (200), (220), and (311) from the planes of crystalline gold (Figure S3). The silver NPs (Figure S3) also exhibited similar crystallographic reflections of Bragg’s peaks centred at 38.117, 44.278, 64.427, and 77.475 (2θ). The peaks matched for the face-centred cubic crystalline metallic gold and silver phases, as documented by the Joint Committee on the Powder Diffraction Standards (JCP2 gold number 04-0784 and JCP2 silver number 04-0783, respectively). The results also corroborated the non-agglomerated nature of the synthesised NPs, as seen on the TEM micrographs and the bright circular ring of the SAED (Figure 3). The extra peaks observed in the GR-AgNPs was presumably due to contamination by silver chloride (AgCl) during the sample preparation.

### 2.6. Attenuated Total Reflection Fourier-Transform Infrared

**Figure 4:**
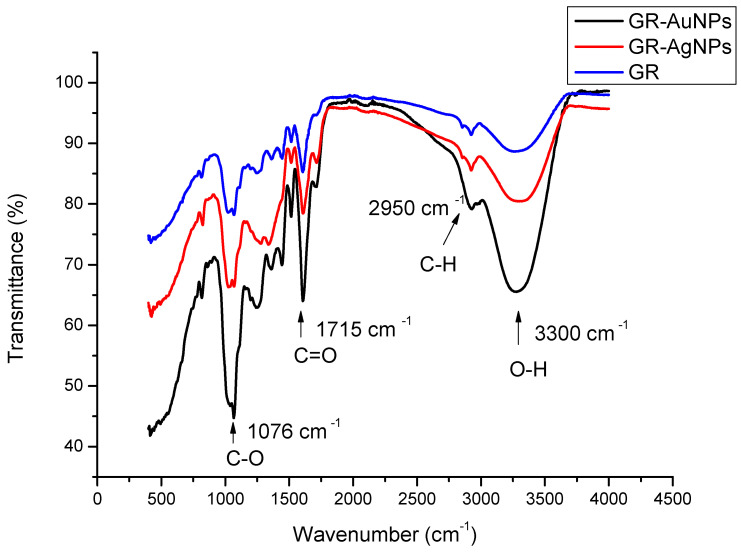
FTIR spectra of the GR, GR-AuNPs, and GR-AgNPs.

**Figure 5:**
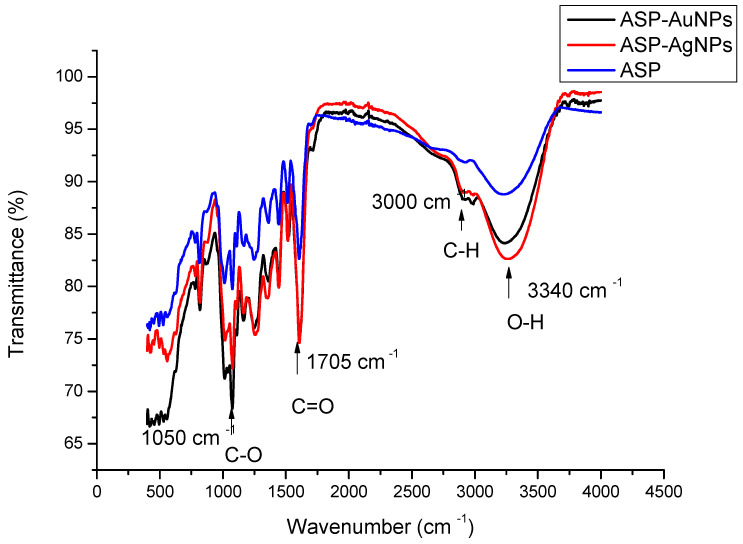
FTIR spectra of the ASP, ASP-AuNPs, and ASP-AgNPs.

*A*. *linearis* contains numerous polyphenols, with aspalathin having a structure in which the aromatic ring is linked to O-H groups, C-O, and α, β-unsaturated ketone. These functional groups in many teas and herbal tea plants were reportedly involved in the synthesis of gold nanoparticles using black tea made from *Camellia sinensis* [27]. A comparison of the FTIR of the GR and ASP and their synthesised nanoparticles showed that the major peaks located at 1050–1076, 1698–1715, 2950–3000, and 3300–3340 cm^−1^ corresponded to their respective C-O (phenolic), C=O (α, β-unsaturated ketone), C-H (aromatics), and a broad O-H vibrational stretching bonds [23]. According to the IR spectra, the functional groups present in GR and ASP were also found in both AuNPs and AgNPs. The C-H vibrational frequencies of ASP in the AuNPs and AgNPs were shifted from 2995 cm^−1^ to 3000 cm^−1^ in the two NPs. Additionally, the C-O bands of GR and the synthesised NPs were blue-shifted by approximately 3 cm^−1^ in the NPs. The spectra also showed higher intensities of the peaks for all the NPs before synthesis (Figure 4 and Figure 5). These changes in the absorptions before and after synthesis were indicative of their involvement in the bio-reduction process [17].

### 2.7. In Vitro Stability Study

**Figure 6:**
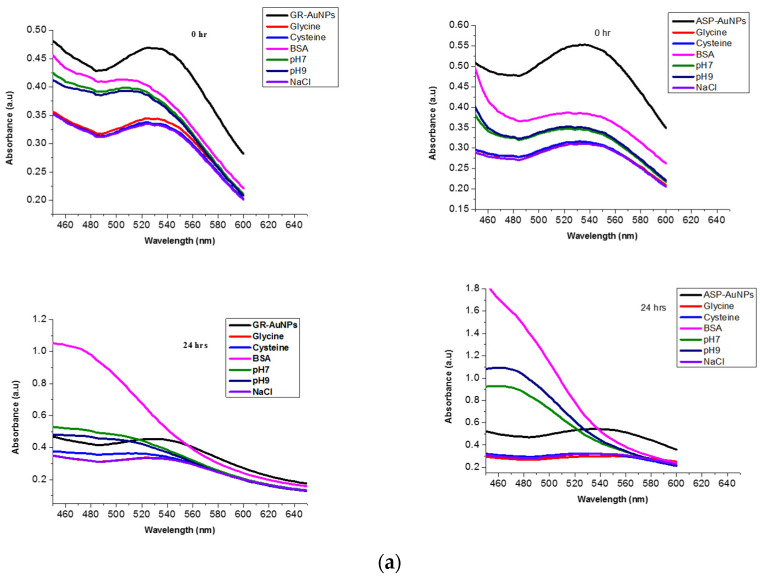

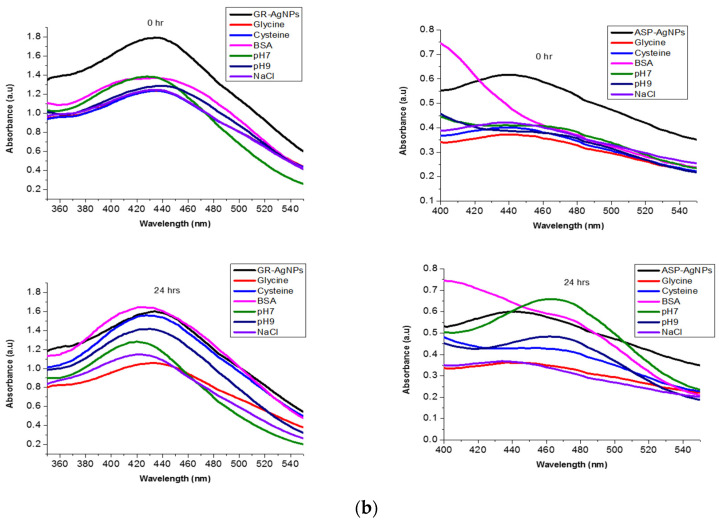
(**a**) In vitro stability of GR and ASP-AuNPs in biogenic media. (**b**) In vitro stability of GR and ASP-AgNPs in biogenic media.

**Figure 7:**
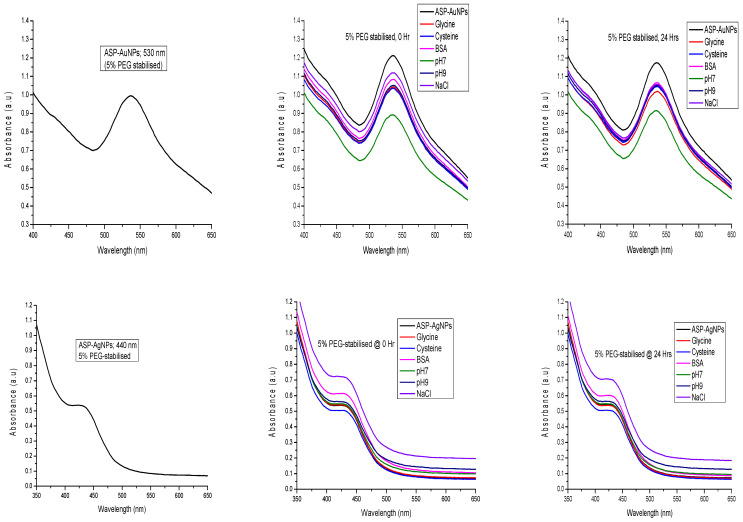
In vitro stability of PEG-stabilised ASP-AuNPs and ASP-AgNPs in biogenic media.

The in vitro stability study was performed for the prepared Au- and AgNPs in different biogenic media. The synthesised Au- and AgNPs were mixed with (0.5%) BSA, glycine, cysteine, (1%) NaCl solution, and 50-mM PBS at pH 7 and pH 9 in a 96-well plate in a ratio of 2:1. The ASP-AgNPs, ASP-AuNPs, GR-AuNPs, and GR-AgNPs without media were used as the controls. The mixtures and the controls samples were then incubated, and the absorbance measurements were monitored on a UV–Vis spectrophotometer at 0 h and 24 h. A 24-h interval was selected to reflect the approximate time required for the complete biodistribution of drugs in the physiological systems. Stable NPs are expected to maintain nearly the same wavelengths as the control samples within a certain time interval before becoming unstable. Figure 6a,b revealed stable absorption bands for GR-AgNPs at 440 nm within the time interval. However, the ASP-AgNPs, ASP-AuNPs, and GR-AuNPs were unstable, showing different absorption maxima within these time intervals. Interestingly, the resynthesis of ASP NPs using 5% polyethene glycol as a stabiliser afforded stable ASP-AuNPs (530 nm) and ASP-AgNPs (440 nm) within 0–24 h in all the tested media (Figure 7).

### 2.8. Cell Viability with Biosynthesised AgNPs and AuNPs Using GR and ASP

**Figure 8:**
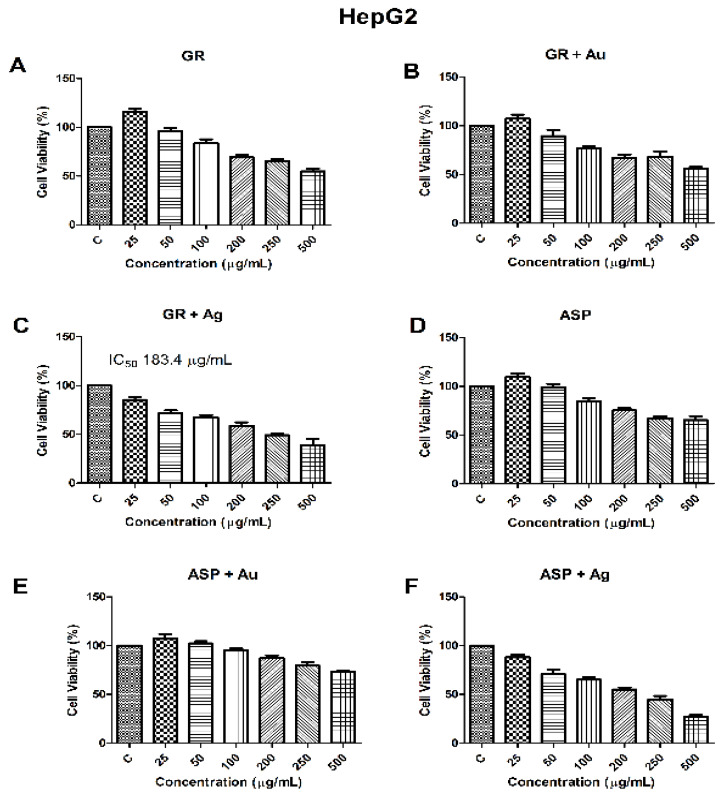
The results of the MTT assay in HepG2 treated with GR total extract (**A**), GR-AuNPs (**B**), GR-AgNPs (**C**), ASP (**D**), ASP-AuNPs (**E**), ASP-AgNPs (**F**) after 24 h (results are reported as the viability in comparison with the control samples). Among the tested samples, the IC_50_ concentration (Subgraph **C**) was most cytotoxic at concentrations 50–100 μg/mL.

**Figure 9:**
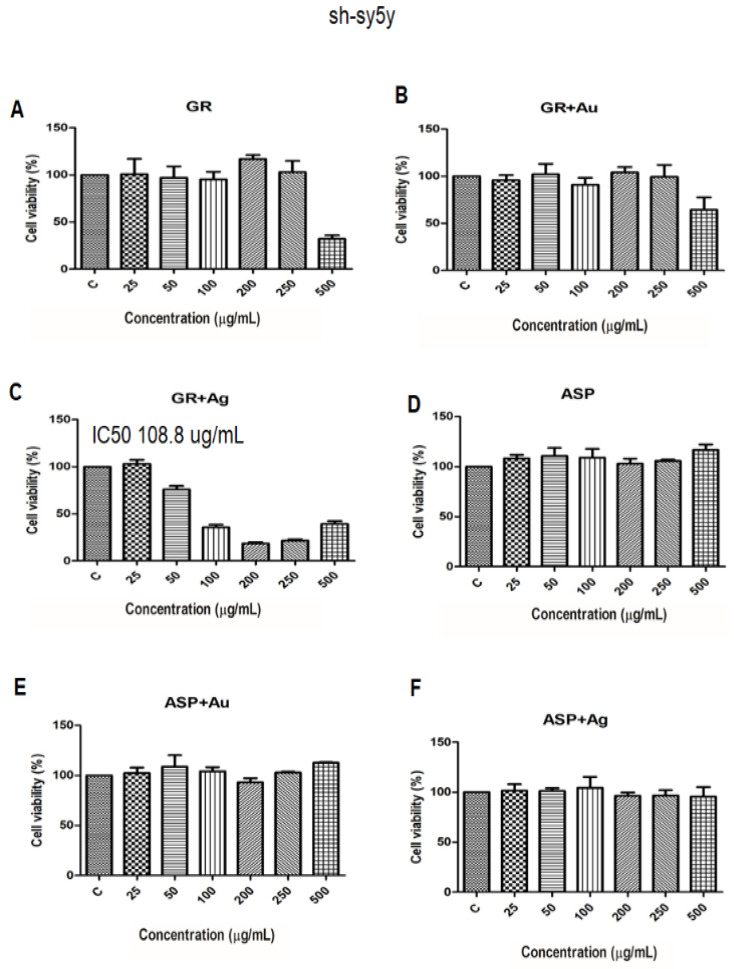
The results of the MTT assay in sh-sy5y treated with GR total extract (**A**), GR-AuNPs (**B**), GR-AgNPs (**C**), ASP (**D**), ASP-AuNPs (**E**), ASP-AgNPs (**F**) after 24 h (results are reported as the viability in comparison with the control sample). Among the tested samples, the IC_50_ concentration (Subgraph **C**) was most cytotoxic at concentrations 50–100 μg/mL.

The 3-(4,5-dimethylthiazol-2-yl)-2,5-diphenyl-2H-tetrazolium bromide assay (MTT) is a conventional method used to determine the viability of metabolic activity in vitro. Both the HepG2 (human liver hepatocellular carcinoma) and SH-SY5Y (human neuroblastoma) cells were treated with 25, 50, 100, 200, and 500 μg/mL of synthesised GR and ASP nanoparticles for 24 h, respectively. The highest activity against the HepG2 cells was observed at the 500 μg/mL concentration for all tested treatments. However, the lowest concentration of 25 μg/mL showed the least antiproliferation effect, which was comparable to the control group. From the results of the cell viability (Figure 8) with synthesised MNPs, it was evident that the GR silver nanoparticles were the most effective against hepatocellular carcinoma, as it was the only nanoparticle that was able to decrease the cell viability to below 50% at concentrations higher than the determined IC_50_ (184.4 µg/mL). A similar trend was observed in the neuroblastoma cell line (Figure 9), as we observed that only silver GR nanoparticles were able to induce a 50% loss of the cell viability (IC_50_ = 108.8 µg/mL). However, this cell line was less sensitive to all other nanoparticles when compared to the HepG2 liver cells, as the cell viabilities remained comparable to the untreated cells.

### 2.9. Cellular Uptake of AgNPs and AuNPs from GR and ASP

**Figure 10:**
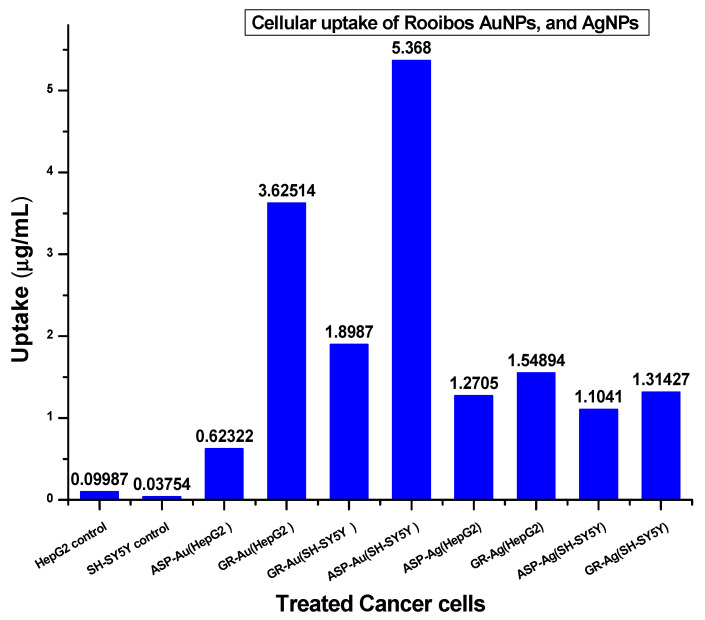
ICP-OES measurements of the cellular uptake of GR/ASP AuNPs and AgNPs.

The cellular uptake of the metallic nanoparticles used as drug carriers plays an important role in atom economy, dose loading, and their interactions with physiological media. To confirm the uptake of nanoparticles of both gold and silver, an ICP-OES analysis on each cell treated with 100 μg/mL each of AuNPs and AgNPs was carried out using standard solutions of each metal for determination of the standard curves for each metal. Figure 10 shows the concentrations of the AuNPs and AgNPs of rooibos assimilated in the tested cancer cells. The uptake of the ASP-AuNPs (5.368 μg/mL) and GR-AuNPs (3.625 μg/mL) by SH-SY5Y and HepG2 cells were relatively higher than their corresponding AgNPs. However, the uptake of lower concentrations of GR-AgNP (1.549 μg/mL and 1.314 μg/mL) by HepG2 and SH-SY5Y, respectively, further supported the observed anticancer activities in the MTT assay at lower concentrations. It is well-known that AuNPs perfectly penetrate cells, especially cancer cell lines [28,29], and this may help in the treatment compared to using Ag. The high penetration of Au may represent another concern if we consider the neuroblastoma cells; however, further work is required in this regard. These results also suggest that a higher dose of AgNPs on cancer cells could lead to clogging of the interface between the cells and the active functional groups on the NP surfaces, causing low permeation into the cells.

## 3. Discussion

**Figure 11:**
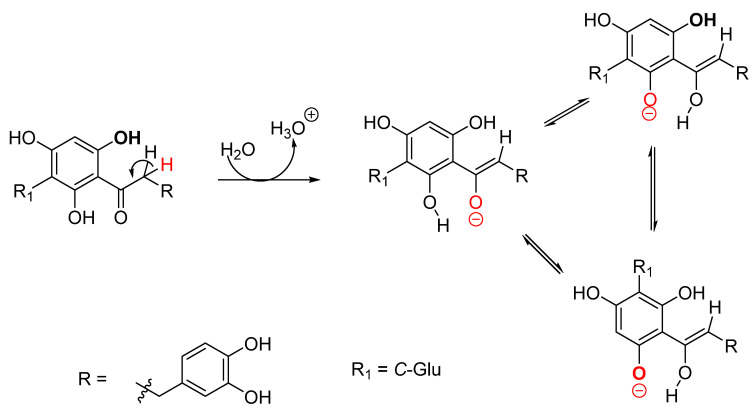
The proposed reduction mechanism of aspalathin.

The chemistry of GR and ASP are interwoven, as GR remains the only source of ASP until the time of this report. Other phenolic constituents of interest in GR are orientin, iso-orientin, and nothofagin [30]. The chemical structure of ASP (Figure 1) makes it prone to oxidation during incubation due to its phenolic nature [11]. This characteristic makes it suitable for use as a reducing agent that could bring out the reduction of metals in a solution to a stable zero valence state. The polyphenols provide electron donors for the reduction/scavenging of free radicals via the hydroxyl (2′, 4′, and 6′) and carbonyl functional groups in the AC ring (Figure 1). The 3,4 dihydroxy derivatives are relatively less active due to strongly bonded phenoxyl groups [31]. The 2′, 4′, 6′-trihydroxy and carbonyl groups present in ASP have also been attributed to the potent antioxidant activities of phloretin and 2,4,6-trihydroxy acetophenone, which have similar dihydrochalcone skeletons [32]. Rezk et al. (2002) showed that the antioxidant/reducing ability of phloretin involves a keto–enol transformation of the carbonyl group and the abstraction of the α-hydrogen atom of the carbonyl group to produce oxygen radicals (Figure 11).

The IR data (Figure 4 and Figure 5) suggest the presence of hydroxyl (3340 cm^−1^), carbonyls (1710 cm^−1^), and alcohols (1050 cm^−1^), which undergoes a slight band shift and intensity. Both GR and ASP demonstrated reducing properties in the synthesis of both Au and Ag metal nanoparticles. The successful synthesis of Au^0^ and the Ag^0^ from phytochemicals of rooibos and their cytotoxic potencies were validated in vitro. The effect of the resonance and absorption of surface electrons of GR and ASP-synthesised Au- and AgNPs showed characteristic absorption peaks at 530 and 440 nm for AuNPs and AgNPs, respectively. The GR-mediated AuNPs and AgNPs (Figure 2a) agree with reports in the literature for these metals [9,21]. A similar result of AuNPs was obtained by a reduction of NaAuCl_4_ with black tea [27] and by the green synthesis of chalcone-capped gold nanoparticles from *Helichrysum foetidum* [17]. In Figure 2b, the AgNPs of ASP exhibited similar absorption peaks at 540 nm (for AuNPs) and 444 nm (AgNPs). From the literature search, and until this study, ASP-mediated AuNPs and AgNPs have been scarcely reported. However, the strong relationship observed between the UV absorption of ASP NPs and GR NPs for each metal further support our results, as ASP represents the major compound found in GR. It is worth mentioning that the GR NPs of Au and Ag show absorptions at lower wavenumbers and at faster reaction times compared to the NPs formed by ASP. These differences in the absorption wavelengths and reaction times in the GR could be due to the synergies of reducing activities contributed by other phytochemicals.

The crystal structure and morphology of the GR and ASP NPs support the feasibility of the biogenic synthesis of MNPs from bioactive compounds. Clear distinguishable particles for each GR and ASP that were mostly spherical were seen under HR-TEM (Figure 3), which is a common shape mostly reported for the plant-based green synthesis of MNPs [8]. In addition to spherical shapes, a rarely reported shape from plant-based MNPs was (hydra-like shape) observed for synthesised GR-AuNPs. The XRD pattern of NPs of the GR and ASP samples further complemented the observed shapes as non-amorphous, and a crystalline signature for all samples were detected. The four main rings for each GR and ASP MNP were indexed as (111), (200), (220), and (311) reflections of the cubic face centre matching the face-centred cubic crystalline metallic gold and silver phases, as documented by the Joint Committee on Powder Diffraction Standards (JCP2 gold number 04-0784 and JCP2 silver number 04-0783, respectively).

The mean particle sizes and colloidal stability (zeta potentials) of metal nanoparticles for use as drug candidates are among essential considerations for efficient biodistribution and clearance in the cytosol [33]. The HR-TEM images of GR and ASP-mediated AgNPs from the statistical analysis exhibited a smaller size range (6.7 ± 0.4 nm to 1.6 ± 0.08 nm, respectively) than was reported for GR-AgNPs in the literature [9]. On the other hand, GR and ASP-mediated AuNPs displayed mean particles sizes (7.5 ± 0.34 nm and 12.5 ± 0.04 nm, respectively) that were very close to the values reported for GR-AuNPs [21]. As observed (Figures S1 and S2), the zeta potential values of both GR and ASP MNPs indicated that the interactions of the NPs and aqueous media led to high negative zeta potentials due to interparticle repulsion forces, such that they repelled each other and had no tendency toward particle aggregation. However, in vitro stability studies of ASP NPs (without PEG) in biogenic media proved contrary to its hydrodynamic stability, especially in neutral-to-alkaline pH (Figure 6). Notable is the instability of ASP NPs at pH ≥ 7 and in BSA. ASP is relatively acidic and was reported to be stable in PBS at pH 3 for up to 29 h and less stable at pH 7 [34]. This agrees with the pH-dependent stability of aspalathin previously reported [34]. The authors showed that ASP was unstable at pH above 7.4, transforming to dihydro-iso-orientin and, further, to iso-orientin and orientin [30,35]. The instability in BSA is presumably due to its complex mix of amino acids and forces interfacing with ASP, which result in conformational changes of the BSA [36]. The stability of ASP in these media was improved by using 5% PEG as both the stabiliser and surfactant during synthesis. We hypothesised that the 5% PEG elicited a lowering effect on the surface tension between the suspension of ASP MNPs and a solution of PBS ≥ 7 and BSA to the stabilised ASP AuNPs and AgNPs (Figure 7).

The cytotoxic effects of various extracts from rooibos using several in vivo and in vitro cancer models have been reported [14,37,38,39,40]. However, there are limited studies on the synthesis of nanoparticles using green rooibos or its unique dihydrochalcone aspalathin and the evaluation of its cytotoxic effects. The results of the MTT assay showed the nontoxic effects of both the ASP and total extract, while the increase in the concentrations of the GR- and ASP-AgNPs enhanced the inhibitory effect on the cell proliferation in the HepG2 and SH-SY5Y cell lines. The IC_50_ value of the GR-AgNPs (183 and 108 μg/mL, respectively) demonstrated that a lower concentration of AgNPs was required to inhibit cell growth than the intact total extract of GR or pure ASP. A putative mechanism of action of the silver nanoparticles could be by an inducement of a state of oxidative stress on the cells by generating free radicals and reactive oxygen species (ROS) through disruption of the mitochondrial function [41]. ROS have been associated with various processes such as DNA and cellular macromolecule damage, interference with cellular signalling cascades, and the initiation of apoptotic processes. Although the exact cellular uptake mechanisms by which GR- and ASP-AgNPs have not been studied will need further investigation, it is plausible that the above-mentioned pathways are activated. 

The cellular recognition of NPs for uptake is known to be influenced by the physicochemical properties of the particle, such as shape and size and the capping agent attached to the NPs [42]. Successful interactions of NPs with the cell membrane could lead to penetration through any of the following mechanisms: phagocytosis, micropinocytosis, endocytosis, diffusion, or adhesion [42]. According to Meiyu et al., smaller-sized Ag-NPs can permeate the cell membrane relative to bigger-sized NPs [43]. An analysis of the ICP-OES quantification shows that the membrane crossing of GR- and ASP NPs was not size-dependent. The uptake was in decreasing order of the ASP-AuNPs (SH-SY5Y) > GR-AuNPs (HepG2) > GR-AuNPs (SH-SY5Y) > GR-AgNPs (HepG2). Although the Ag-NPs of both samples were smaller in size when compared to the Au-NPs, a higher concentration of Au-NPs were taken up by both neuroblastoma and hepatocellular cancer cells (Figure 10). The observed gold uptake can be attributed to its optical property, inertness, high biocompatibility and bioconjugation, and low toxicity in biomolecules [44].

### Proposed Mechanism of the Metal Nanoparticles Formation

**Scheme 1:**
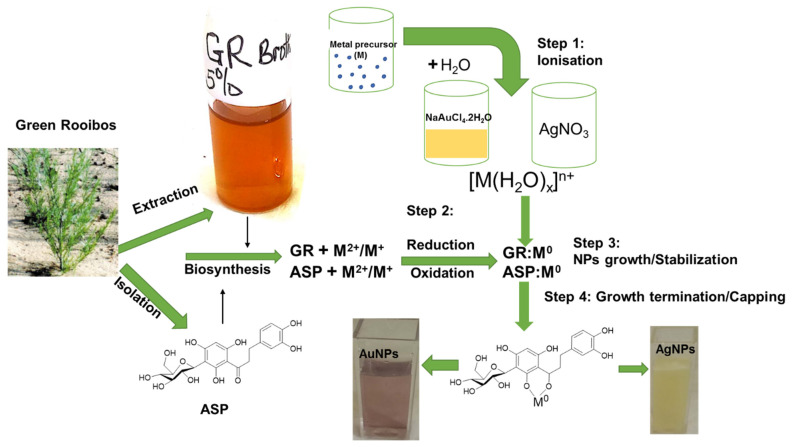
Proposed mechanism of synthesis of gold and silver nanoparticles with GR or ASP and a gold precursor.

The chemical structure of aspalathin (ASP) shows the properties of a typical Lewis base due to the availability of a divalent oxygen atom in the polyphenols. The electron-deficient M^n+^ ions in the solution could give stable nanoparticles, with aspalathin containing oxygen radical/donors groups. Scheme 1 (and Figure 11) explains our proposed reaction mechanism in an aqueous mixture of both GR and ASP with Au^3+^/Ag^+^ ions from sodium tetrachloroaurate (III) dihydrate (NaAuCl_4_·2H_2_O)/silver nitrate (AgNO_3_). We propose a four-step reaction involving; (i) ionisation of the metal precursor, (ii) reduction the of Au^3+^/Ag^+^ ions to their respective M^0^ through electron donation by GR-containing molecules and ASP (Figure 11), (iii) NP growth/stabilisation by dative π-bonding of donated electrons, and (iv) growth termination/capping. ASP is the major bioactive constituent of GR; it behaves as the oxidising agent for M^n+^ cations. Additionally, the oxidising ability of ASP is easily noticed because of the high stability of the formed quinoid structure after donating electrons (Figure 1 and Figure 11 and Scheme 1). This increases the tendencies for Au^3+^/Ag^+^ to be reduced to neutral Au^0^/Ag^0^. The stabilisation of the zerovalent oxidation state of Au^0^/Ag^0^ by the capping agent(s) at the surface can be attributed to the available *d*-orbital space for dative π-bonding with lone pairs of electrons from the O=C carbonyl and O-H groups and/or the protonated positively charged OH groups attracted by the electron cloud and the surfaces of the MNPs, as explained before [17]. This proposed mechanism is further supported by the band maxima of the GR-AuNPs, GR-AgNPs, ASP-AuNPs, and ASP-AgNPs at 530, 440, 540, and 44 nm, respectively, which can be attributed to the ligand-to-metal charge transfer.

## 4. Materials and Methods

### 4.1. General

Freshly dried and pulverised green rooibos plant material was generously donated by the South African Rooibos Council, while pure aspalathin was isolated in house. Analytical grade metal salt and acid were used as precursors. Sodium tetrachloroaurate (III) dihydrate (NaAuCl_4_·H_2_O, 99.99%), the gold standard, silver nitrate salts, silver standard and polyethene glycol was supplied by Sigma-Aldrich (Cape Town, South Africa), while Milli-Q deionised water used was produced in house.

### 4.2. Cell Culture and Cell Conditioning

Liver and neuroblastoma cell culture reagents were purchased from Thermo Fisher. All other consumables were purchased from Sigma Aldrich (St. Louis, MO, USA). The HepG2 cells were cultured in minimum essential media (10% foetal calf serum, 1% penstrepfungizone, and 1% L-glutamine) at 37 °C in a humidified incubator (5% CO_2_). Cells were allowed to reach 80% confluency in culture flasks before treatment with the full rooibos extract and aspalathin nanoparticles. On the other hand, the neuroblastoma cells were cultured in Dulbecco’s minimum essential media (10% foetal calf serum, 1% penstrepfungizone, and 1% L-glutamine) at 37 °C in a humidified incubator (5% CO_2_). Cells were allowed to reach 80% confluency in culture flasks before treatment with the total green rooibos extract and aspalathin nanoparticles. The two cell lines were then treated for 24 h with a range of concentrations (0–500 μg/mL) of both rooibos nanoparticles mentioned before the experimental analysis was conducted.

### 4.3. Experimental

#### 4.3.1. Preparation of Plant Extract and Isolation of Aspalathin

Five grams of freshly dried and pulverised green rooibos (GR) plant materials (consisting mainly of leaves and stems) were extracted in 100-mL deionised water heated to 70 °C for 40 min, resulting in a reddish-brown aqueous extract. The freshly prepared aqueous extract (5% GR) was centrifugated at 800 rpm for 20 min, filtered with a 0.45-μm syringe filter, stored at 4 °C, and used within two days of preparation. Details for the purification of aspalathin was reported earlier [20].

#### 4.3.2. Biogenic Synthesis of Gold and Silver Nanoparticles

The synthesis of nanoparticles of the metals required six different set-ups. Hence, a two-batch experiment was carried out. The first batch (Batch A) involved the stepwise addition of the aqueous extract of GR (acting as a reducing/capping agent) to solutions of each metal precursor separately, while, in the second batch (Batch B), the purified compound ASP was used in place of the GR. In a typical experiment, 10 mL of the aqueous GR extract (Batch A) was added separately and in a dropwise manner to a 90-mL heated (at 70 °C) solution of 0.001 M of each of the metal nanoparticle precursors, stirring under reflux. The resultant mixtures were allowed to react until a visible colour change was observed. The colour change was usually an indication of complete biosynthesis. A change to a ruby red colour (28 min) for AuNPs from the initial pale-yellow colour was noticed. Additionally noticed was the appearance of a yellowish-brown colour (30 min) for AgNPs from a colourless AgNO_3_. These procedures were repeated for the batch B experiment, in which 20 mg of ASP dissolved in 2 mL of deionised water were added to each metal solution in place of the total green rooibos extract. A similar colour change observed in batch A was noticed with a time difference of ±10 min for the reaction times.

#### 4.3.3. Characterisation of Nanoparticles

The structural measurements of the nanoparticles were carried out using spectroscopic and imaging techniques, including ultraviolet–visible light (UV/Vis), high-resolution transmission electron microscope (HR-TEM), X-ray diffraction (XRD), Fourier-transform infrared (FTIR), and dynamic light scattering (DLS) measurements. The surface plasmon resonance absorption peak characteristic of metal nanoparticles was monitored by a polar star Omega microlitre plate UV/Vis reader (BMG Labtech, Ortenberg, BW, Germany). Characteristic functional groups involved in the synthesis were recorded on a PerkinElmer Fourier-Transform Infrared Spectrometer 2000 equipped with a universal ATR-FTIR (PerkinElmer Spectrum 100, Llantrisant, Wales, UK) in the range of 400–4000 cm^−1^. The morphology of the biosynthesised AuNPs and AgNPs were studied with High-Resolution Transmission Electron Microscopy (FEI Tecnai G2 F20 S-Twin HRTEM, operated at 200 kV). For each NP, a few drops were placed on a carbon-coated copper grid and allowed to dry completely at room temperature. The crystal structures of the samples were then determined by X-ray diffraction (X-ray diffraction Model Bruker AXS D8 advance with a copper-sealed X-ray source producing Cu *k*α radiation at *k*α_1_ = 1.5406 Å). The sample was mounted in the centre of the sample holder on a glass slide and levelled to the correct height. The measurements were performed within a range in 2θ between 30° and 80° with a typical step size of 0.034° in 2θ. A position-sensitive detector, Lyn-Eye, was used to record the diffraction data at a typical speed of 0.5 s/step, which is equivalent to an effective time of 92 s/step for a scintillation counter. The analysis of the HR-TEM and XRD images was carried out using ImageJ software, 1.50b version 1.8.0_60 (http://imagej.nih.gov/ij, accessed on 23 July 2021) and Origin pro-2019 (64 bits) software, respectively. 

#### 4.3.4. Stability Study

The stability of the synthesised NPs in the solution was studied using two techniques, the electrophoretic light scattering (ELS) based on the Smoluchowski theory and in vitro stability in biogenic media at a given time interval. The ELS technique provides zeta potential, which indicates the potential stability of the particles in a colloidal system. This was done using a Malvern Zetasizer Instrument with Zetasizer software version 7.11 (Malvern Ltd., Worcestershire, UK) at a 25° and 90° angle. The in vitro stability test was carried out by mixing 200 μL of gold nanoparticles solution with 100 μL of biogenic amino acids (such as 0.5% glycine and 0.5% BSA), phosphate-buffered saline (PBS at pH 7 and pH 9), and 1% NaCl solution in a 96-well plate and incubated (at 37 °C) for different periods. The stability of the AgNPs and AuNPs in these media were monitored with UV–Vis based on the retention of their absorbance maxima at an interval of 6 h.

#### 4.3.5. Cell Viability

The MTT assay was extensively used for determining the cytoprotective effects of several phytochemicals [45]. To monitor the cell viability as a measure of the metabolic activity, the MTT assay was performed. After the 24-h exposure, the HepG2 and SH-SY5Y cells (15,000 cells per well in 96-well plates) were incubated for 2 h with 100 μL of MTT solution (5 mg/mL) after PBS washing. The MTT formazan crystals were then solubilised by DMSO resuspension. The optical density of the formazan product was read at a 570-nm wavelength with a reference wavelength of 690 nm using a multi-plate reader (Multiska Thermo Scientific, version 1.00.40, Waltham, MA, UK. The half-maximal inhibitory concentration (IC_50_) was determined. The results were analysed using GraphPad Prism5. The IC_50_ value defined the potency of a substance in inhibiting a specific biological or biochemical function. For the nanoparticles, it was the concentration at which 50% of the cells died.

#### 4.3.6. In Vitro Cellular Uptake of Au and Ag Nanoparticles

The cellular uptake of AuNPs and AgNPs have been studied by different methods that can be classified as either qualitative or quantitative methods [46,47]. Qualitative methods measure the response of treated cell MNPs by colorimetry, fluorescent changes, or by visualisation of the location of the metal in treated cells via transmission electron microscope or an energy dispersive X-ray (EDX) analysis. Although there is yet to be a generally accepted standard for the quantification of MNP entry into living cells due to the limitations posed by different physicochemical properties of the particles and types, biological media, etc., a quantitative method reportedly used to determine the concentration of MNPs taken up by physiological media involves the use of inductively coupled plasma-(mass spectroscopy/optical emission spectroscopy), ICP-MS, or ICP-OES within a selected range of standard solutions of the metal [44]. For the cellular uptake of our AuNPs and AgNPs, an in-house protocol was used. Briefly, 1 × 10^5^ of HePG2 and SH-SY5Y cells treated with (GR and ASP) AuNPs and AgNPs were seeded in 6-well plates and incubated for 24 h. After incubation, the medium was discarded, and the cells were washed with PBS. The cells were trypsinised into 15-mL tubes, centrifugated at 3000 rpm, rewashed with PBS, and centrifugated again. The PBS was discarded, and the MNPs were digested with aqua regia (1 mL) at 80 °C for 2 h to release the metal ions. The released metal ions from the cells were then quantified with the ICP-OES analysis. In the ICP-OES analysis, standard solutions each of gold and silver of 100 ppm were prepared, and serial dilutions of these concentrations gave 50 ppm, 25 ppm, 12.5 ppm, and 6.25 ppm, which were used for the standard calibrations. The uptake of the MNPs by cells corresponded to the concentrations of the gold ions and silver ions in the cells.

#### 4.3.7. Statistical Analyses 

The biological experiments were conducted in triplicate (independently). Statistical data were analysed by one-way ANOVA and the Bonferroni test for multiple group comparisons, unless otherwise stated. The software used for all analyses was GraphPad Prism v. 5.0 software (GraphPad Software Inc., San Diego, CA, USA). The results were considered statistically significant when *p* < 0.05.

## 5. Conclusions

This study contributed to the biosynthesis of metallic nanoparticles for biomedical applications. It showed that the use of the aqueous extracts of *Aspalathus linearis* (green rooibos) and aspalathin were effective in the bio-reduction of gold and silver crystalline nanoparticles. Additionally, the study showed that the pure compound had a comparative advantage for smaller sizes of nanoparticle formations from the total plant extracts. Furthermore, the use of silver nitrate precursors could elicit smaller sizes for both GR and ASP nanoparticles over the gold precursor. Based on the cytotoxic potency, the comparative analysis of the IC_50_ values of the parent GR and ASP and the synthesised silver nanoparticles of GR and ASP indicated that the synthesised silver nanoparticles were more efficacious in preventing cancer cell growth compared to the plant extract or the pure compound alone. The combined results of this study confirmed that the pure compounds had the reducing/capping abilities for the synthesis of metallic nanoparticle and could potentially be applied in biomedical fields.

## Figures and Tables

**Table 1:** DLS analysis, particle sizes, zeta potential, and polydispersity index of the MNPs under study.

Samples	Hydrodynamic Sizes (nm)	Zeta Potential (mV)	PDI
GR-AuNPs	107.0	−26.2	0.128
GR-AgNPs	109.0	−15.0	0.274
ASP-AgNPs	136.4	−26.7	0.100
ASP-AuNPs	104.7	−19.2	0.111

## Data Availability

The raw data presented in this study are available on request from the corresponding author.

## Supplementary Materials

The following are available online at https://www.mdpi.com/article/10.3390/plants10112460/s1: Figure S1: Zeta potential; ASP-AuNPs (I), GR-AgNPs (II), GR-AuNPs (III) and ASP-AuNPs (IV), Figure S2: Hydrodynamic Average size; ASP-AuNPs (I), GR-AgNPs (II), GR-AuNPs (III) and ASP-AuNPs (IV), Figure S3: XRD patterns showing face-centred cubic phases of GR and ASP AuNPs and AgNPs.
