# Supplementary material for: Cytotoxic Effects of Phytomediated Silver and Gold Nanoparticles Synthesised from Rooibos (Aspalathus linearis), and Aspalathin
Source: Plants (Basel). 2021 Nov 15;10(11):2460. doi: 10.3390/plants10112460 (PMC8620073; doi:10.3390/plants10112460)
Supplement: Supplementary file 1 [file plants-10-02460-s001.zip › plants-1414337-supplementary.pdf]

Cytotoxic Effects of Phytomediated Silver and Gold Nanoparticles Synthesised from Rooibos (*Aspalathus linearis*), and Aspalathin

Akeem O. Akinfenwa [1], Naeem S. Abdul [2], Fathima T. Docrat [2], Jeanine L. Marnewick [2], Robbie C. Luckay [3], Ahmed A. Hussein [1*]

SUPPLEMENTARY DATA

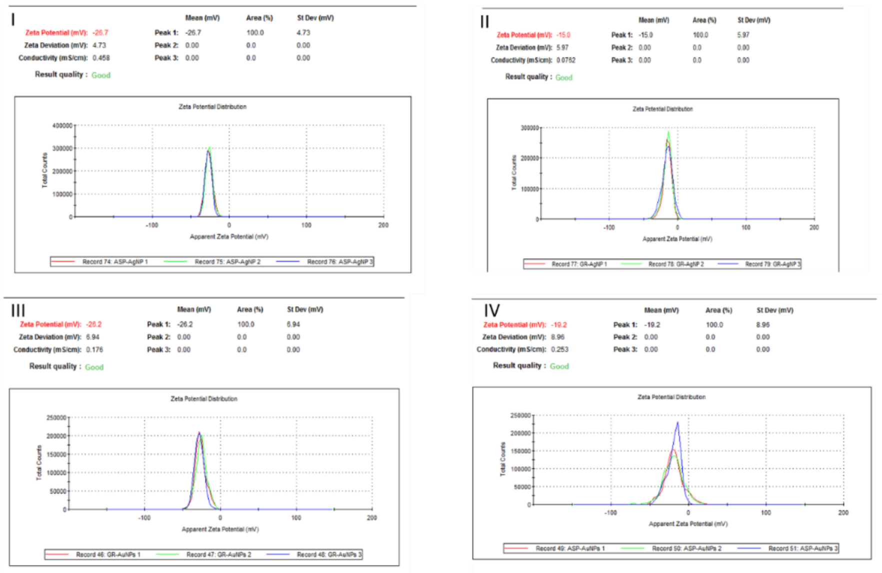

Figure S1. Zeta potential; ASP-AuNPs (I), GR-AgNPs (II), GR-AuNPs (III) and ASP-AuNPs (IV)

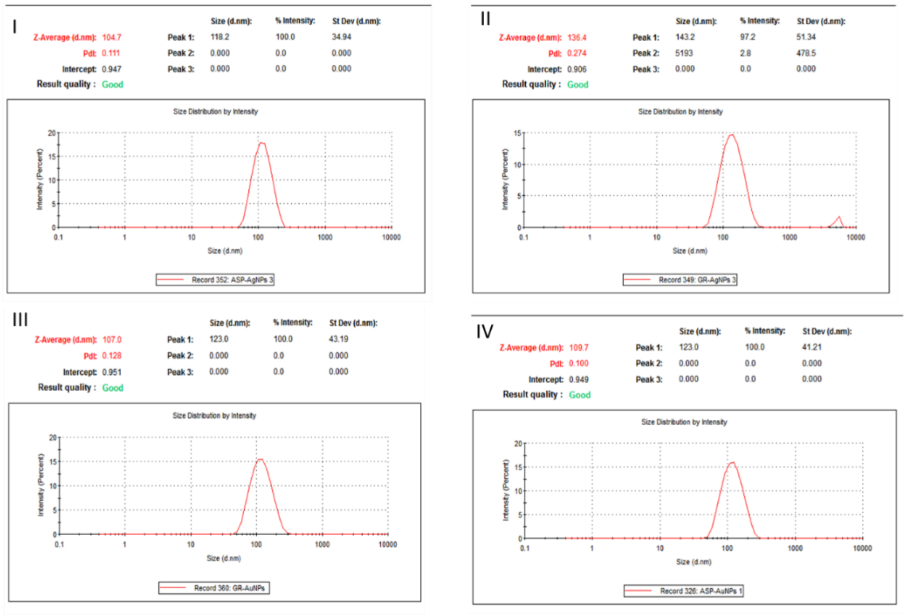

Figure S2. Hydrodynamic Average size; ASP-AuNPs (I), GR-AgNPs (II), GR-AuNPs (III) and ASP-AuNPs (IV)

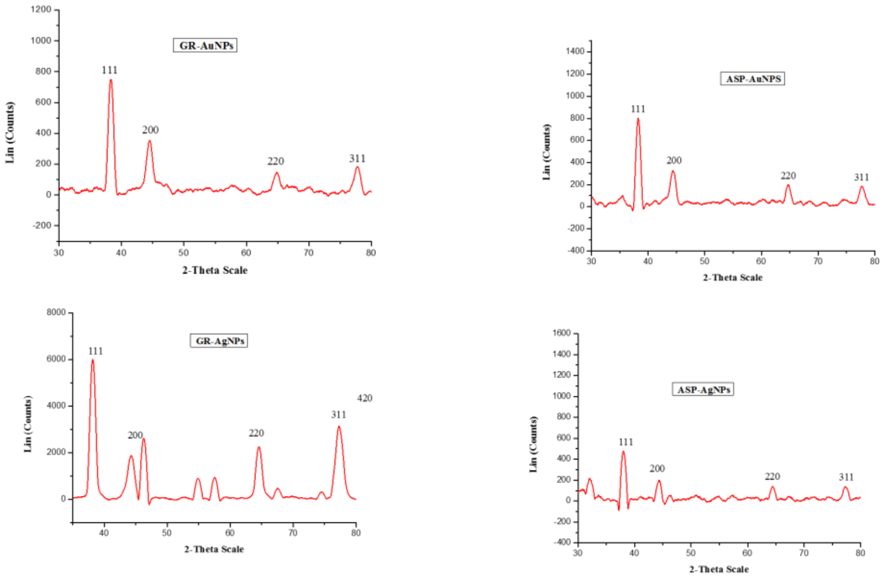

Figure S3 6. XRD patterns showing face-centred cubic phases of GR and ASP AuNPs and AgNPs
